# Analysis of Inbreeding Coefficient and Genetic Diversity in Xinjiang Brown Cattle Based on Pedigree and ROH Evaluation

**DOI:** 10.3390/ani16010042

**Published:** 2025-12-23

**Authors:** Kailun Ma, Xue Li, Yanyan Shang, Jiangjiang Wei, Menghua Zhang, Dan Wang, Xixia Huang, Qiuming Chen, Lei Xu

**Affiliations:** College of Animal Science, Xinjiang Agricultural University, Urumqi 830052, China; makailun0829@163.com (K.M.);

**Keywords:** Xinjiang brown cattle, ROH, inbreeding coefficient, genetic diversity, candidate genes

## Abstract

The Xinjiang Brown cattle (XJBC) is one of China’s five major dual-purpose dairy and beef breeds. Analyzing the genetic diversity of the Xinjiang Brown cattle population lays the theoretical groundwork for identifying and conserving its genetic resources. This study employed the Illumina Bovine SNP 150K chip to analyze genetic diversity, inbreeding coefficient, kinship, and genetic distance in a population of 750 Xinjiang Brown cattle from three breeding farms in Xinjiang. Genetic diversity was assessed by calculating minimum allele frequency (MAF), observed heterozygosity (Ho), expected heterozygosity (He), polymorphic information content (PIC), and linkage disequilibrium (LD). Population structure was analyzed using PCA. ROH was calculated to derive ROH-based inbreeding coefficients, pedigree-based inbreeding coefficients (*F*_PED_) were estimated using CFC software for comparison, and candidate genes within high-frequency ROH regions in Xinjiang Brown cattle were identified. A G matrix was constructed to analyze population kinship. Results revealed 94,173 high-quality SNP loci in Xinjiang Brown cattle, with an average MAF of 0.276, PIC of 0.376, Ho of 0.345, and He of 0.376. Breeding farm 3 exhibited the fastest LD decay, indicating relatively high genetic diversity across Xinjiang Brown cattle populations, with farm 3 demonstrating greater diversity. The IBS genetic distance was 0.313. The G matrix results aligned with the IBS distance matrix, both indicating close kinship among some individuals within the Xinjiang Brown cattle population. The ranges for average *F*_PED_ and average *F*_ROH_ across farms were 0.0017–0.0189 and 0.0609–0.0878, respectively. Short ROH segments (0.5–2 Mb) constituted the largest proportion (51.31%) of all ROHs. Within high-frequency ROH enrichment regions, 61 genes, including *LCORL*, *FAM110B*, *NR4A1*, and *PER2*, were identified as potentially associated with economic traits in Xinjiang Brown cattle. These findings provide relevant marker sites for genomic selection in Xinjiang Brown cattle and lay a theoretical foundation for subsequent research.

## Simple Summary

The Xinjiang Brown cattle is a dual-purpose dairy and beef breed independently developed in China, exhibiting excellent performance in both milk and meat production, and is highly favored by farmers and herders. Genetic analysis was conducted on 750 Xinjiang Brown cattle from three breeding farms using a high-density SNP chip. This study revealed abundant genetic diversity within the population, with breeding farm 3 exhibiting particularly high diversity. However, we also identified closely related individuals and assessed the population’s inbreeding level. By identifying regions of shared genomic segments (ROHs) across numerous individuals, we identified 61 candidate genes (including *LCORL*, *FAM110B*, and *NR4A1*) likely associated with important economic traits in Xinjiang Brown cattle. These findings provide a crucial genetic foundation for conserving Xinjiang Brown cattle genetic resources and will directly support future genotyping-based selection breeding programs.

## 1. Introduction

Xinjiang Brown cattle are a dual-purpose breed for milk and meat. This breed was developed over more than 50 years, beginning in the 1930s. It was created through crossbreeding and improvement, followed by long-term selective breeding. Its maternal lineage is derived from Kazakh cattle, while the paternal lineage includes Swiss Brown, Alatau, and a small proportion of Kostroma cattle. The breed was officially approved and recognized in 1983 [1,2]. Xinjiang Brown cattle are known for their ability to thrive on coarse feed, cold tolerance, strong stress resistance, high milk quality, and excellent meat texture. They are well-suited for mountainous grassland grazing and represent one of the primary breeds in Xinjiang’s cattle industry [3]. Xinjiang Brown cattle are the primary breed for dairy and beef production in Xinjiang. By 2023, the total population of Xinjiang Brown cattle and their crossbred descendants reached approximately 1.9 million. This illustrates their popularity among farmers and herders, as well as their widespread presence in northern Xinjiang [4]. The breeding systems for Xinjiang Brown cattle include both intensive housing and semi-intensive systems that combine housing with grazing. Under intensive housing conditions, Xinjiang Brown cattle achieve an average annual milk yield of 6000 kg, with average milk fat and protein contents of 4.2% and 3.5%, respectively. In semi-intensive systems, the average milk yield during lactation is around 2320 kg [5]. For beef-type Xinjiang Brown cattle, the average daily weight gain exceeds 0.9 kg, with a carcass grading yield of up to 75%. The intramuscular fat content ranges from 6.9% to 7.5% [6].

Traditional genetic structure analysis of populations based on pedigrees may be affected by missing records, making it difficult to draw accurate conclusions [7]. In recent years, with the continuous advancement of molecular genetics, high-throughput sequencing technology, and bioinformatics, second-generation molecular marker technology has become a crucial tool for genetic improvement in livestock and poultry, facilitating SNP-based research on genetic diversity. SNPs exhibit characteristics such as broad distribution, high information content, and ease of detection [8]. Currently, population genetic studies based on SNP sequencing technology have been conducted across multiple cattle breeds, including Xinjiang Brown cattle and Chinese Simmental cattle [9], Liangshan cattle [10], and Yunling cattle [11,12], providing crucial insights into the population structure and genetic diversity of these cattle breeds. The inbreeding coefficient represents the probability that an individual inherits identical alleles from both of its parents [13]. It measures the degree of inbreeding within an individual or population, serving as a crucial parameter in livestock breeding. This metric enables the effective management of breeding strategies within populations, helps maintain genetic diversity, and minimizes the accumulation of homozygous alleles, thereby minimizing their potential negative impacts on livestock performance. Over the past decade, inbreeding coefficients for livestock individuals and populations were calculated based on pedigrees collected within farms, playing a vital role in traditional breeding [14]. However, this method heavily relies on the accuracy and completeness of pedigree information. Its limitations—such as ignoring specific genomic details and failing to capture hidden inbreeding—restrict its accuracy and applicability. With advances in genomic technology, researchers have begun utilizing high-density SNP data to calculate genomic inbreeding coefficients [15,16]. This approach was found to be independent of pedigree completeness or accuracy, avoiding biases from pedigree errors or omissions. Furthermore, by analyzing regions of continuous homozygosity (ROHs) across the genome, it can identify hidden inbreeding and region-specific effects, more accurately reflecting actual inbreeding levels [17]. Currently, many studies utilize population-level ROH islands and individual high-density ROH regions to identify genes associated with significant economic traits in cattle [18,19,20].

This study utilized bovine 150K SNP chip data to investigate Xinjiang Brown cattle from three core breeding farms, assessing population genetic diversity, genetic distance, and kinship matrices to further elucidate population structure and relationships. This study estimated and compared *F*_PED_ and *F*_ROH_ in Xinjiang Brown cattle using pedigree and genomic data. This study identified and annotated candidate genes associated with economic traits in Xinjiang Brown cattle within high-frequency regions of recent shared ancestry (ROH). This study provides relevant trait selection sites for the conservation and utilization of Xinjiang Brown cattle genetic resources, as well as for genomic selection.

## 2. Materials and Methods

### 2.1. Experimental Animals and DNA Extraction

In this study, blood samples of 750 Xinjiang Brown cattle were randomly collected from three main Xinjiang Brown cattle breeding farms in Xinjiang from July 2018 to July 2022. The three pastures are: Xinjiang Yanben Brown Cattle Breeding and Development Co., Ltd. (Ningzhouhu Village, Ershilidian Town, Hutubi County, Changji Prefecture, Xinjiang, China), Tacheng Agricultural and Animal Husbandry Technology Co., Ltd. (Baigetuobie Street, Tacheng City, Tacheng Prefecture, Xinjiang, China), and Yili New Brown Cattle Farm (Tielekala Village, Alemal Town, Xinyuan County, Yili Prefecture, Xinjiang, China). The three pastures were all house-feeding, with suitable temperature and humidity, TMR feeding, free drinking water, and machine milking. 750 Xinjiang Brown cattle were all lactating cows in good health. Blood samples were collected from the tail vein, and 10 mL of blood was collected from each individual and stored in an EDTA anticoagulant tube. Subsequently, it was packed into a sterile 1.5 mL centrifuge tube and stored at −20 °C for genomic DNA extraction. The specific number distribution of cattle sampled in each pasture is shown in Table S1. Three generations of pedigree tracing were carried out on 750 Xinjiang Brown cattle with genotyping, and the pedigree records of 1210 Xinjiang Brown cattle were finally included.

### 2.2. Genotype Data Quality Control

Genotype data quality control for 750 Xinjiang Brown dairy cattle was performed using PLINK V1.90b7 software [21] under the following criteria: individuals were excluded when (1) Individual genotype call rate was less than 95%; (2) Single-nucleotide polymorphism (SNP) call rate was less than 99%; (3) SNPs were located on sex chromosomes. To analyze the characteristics of population genetic diversity, the minimum allele frequency (MAF) and Hardy–Weinberg equilibrium (HWE) tests were performed. The final SNP chip data were annotated against the bovine reference genome using ANNOVAR software v2016-02-01 [22].

### 2.3. Statistical Analysis

#### 2.3.1. Population Genetic Diversity Analysis

To understand the genomic genetic diversity status of the Xinjiang Brown cattle population, genetic diversity analysis was conducted on quality-controlled SNP chip data from Xinjiang Brown cattle. PLINK V1.90b7 software was used to calculate He, Ho, MAF, and PIC.

#### 2.3.2. Genetic Distance Matrix and Phylogenetic Analysis

To assess kinship relationships among individuals, IBS genetic distance and G matrix analyses were performed. The IBS matrix of genetic distances between individuals was calculated using PLINK V1.90b7 software. The G matrix of kinship relationships among individuals was constructed using GCTA v1.94.1 software [23]. Finally, a heatmap was generated using R v4.4.1 packages.

#### 2.3.3. Principal Components and Linkage Disequilibrium

Principal component analysis was performed using PLINK V1.90b7 software, with graphical representations generated in Origin 2024. Linkage disequilibrium (LD) patterns across breeding farms were calculated using PopLDdecay v3.42 software [24]. Perl scripts were used to read LD results and generate LD decay plots across three Xinjiang Brown cattle breeding farms, enabling assessment of genetic diversity within the three breeding fields. The PLINK V1.90b7 software was utilized to calculate the genetic distance matrix for each breeding farm. A perl script was then employed to convert this output into .meg format file. Following this, MEGA v7.0 software [25] was used to select the adjacency method for constructing the phylogenetic tree. The resulting phylogenetic tree was exported in nwk format, and the online tool ITOL (https://itol.embl.de/, accessed on 21 October 2025) was used to enhance the visual presentation of the graph.

#### 2.3.4. Inbreeding Coefficient Based on Pedigree Information

The inbreeding coefficient for 750 Xinjiang Brown dairy cows was calculated using CFC V1.0 software [26], based on three-generation pedigrees. The calculation of inbreeding coefficients based on pedigree information can be expressed using the following formula [27]:

$$F_{X}=\sum (\frac{1}{2}{)}^{n_{1}+n_{2}+1}(1+F_{A})$$

In the formula, *F_X_* represents the pedigree inbreeding coefficient of individual *X* of the Xinjiang Brown cattle breed; *n*_1_ and *n*_2_ denote the number of generations from the sire of individual *X* to the common ancestor and the number of generations from the dam of individual *X* to the common ancestor, respectively; *n*_1_ + *n*_2_ +1 indicates the number of individuals in the path chain connecting the sire, dam, and common ancestor of *X*; *F_A_* denotes the pedigree inbreeding coefficient of the common ancestor *A*.

#### 2.3.5. Inbreeding Coefficient Based on Genomic Information

ROH detection was performed using PLINK V1.90b7 software with a sliding window approach for autosomes. Parameter settings were implemented as described by Yu et al. [28] as follows: (1) Maximum allowed heterozygous genotypes: 3; (2) Maximum allowed missing genotypes: 5; (3) Number of SNPs per window: 50; (4) Window heterozygosity threshold: 0.05; (5) Minimum length: 500 kb; (6) Maximum gap between two adjacent heterozygous genotypes: 100 kb; (7) Minimum of 50 SNPs per 50 kb within ROH; (8) Minimum total of 50 SNPs. Statistical analysis was conducted on the number, length, and distribution of ROHs within the Xinjiang Brown cattle population. The formula for calculating the inbreeding coefficient based on ROH is as follows [29]:

$$F_{ROH}=\frac{\sum L_{ROH}}{L_{genome}}$$

In the formula, *F_ROH_* denotes the genomic inbreeding coefficient of individual *X*; ∑*L_ROH_* represents the total length of all ROH segments in the individual’s genome; *L_genome_* indicates the total physical length of the autosome genome; the reference genome version is ARS-UCD1.2, with an approximate autosome total length of 2489.39 Mb. Visualization was performed using Origin 2024 software.

#### 2.3.6. High-Frequency ROH Candidate Regions and Enrichment Analysis

This study employed the detectRUNS toolkit [30,31] within the R environment to statistically analyze the frequency of each SNP locus within ROHs across the Xinjiang Brown cattle population. Regions with a frequency of ROH occurrence ≥ 0.2 were identified by calculating the ratio of each SNP’s contribution to ROH formation against the total sample size, and these were categorized as high-frequency ROH regions. Gene annotation for these overlapping window SNPs was performed using Bedtools software [32] based on the bovine reference genome (ARS-UCD 1.2) and annotation files. Functional information for these genes was queried using NCBI databases and existing literature. To better understand the molecular functions of candidate genes, GO enrichment analysis was performed using the DAVID online portal (https://davidbioinformatics.nih.gov/, accessed on 28 October 2025). *p*-values < 0.05 indicated significant enrichment. Visualization of results was conducted using the MicroBioinformatics online platform (https://www.bioinformatics.com.cn/, accessed on 28 October 2025).

## 3. Results

### 3.1. Analysis of Genetic Diversity in the Xinjiang Brown Cattle Genome

**Figure 1:**
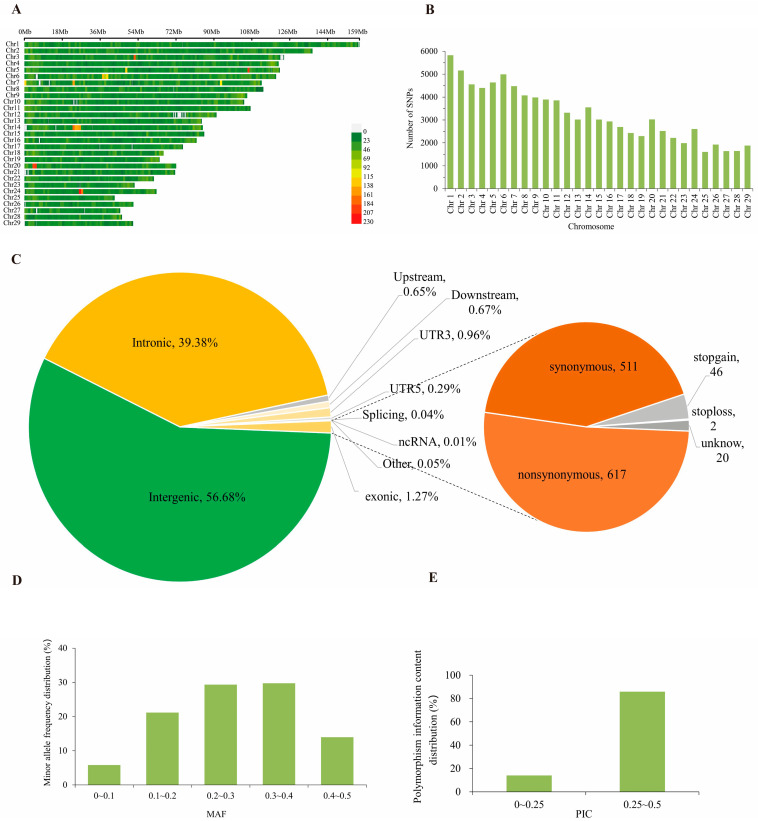
Results of genomic genetic diversity analysis in Xinjiang Brown cattle. (**A**) Distribution of SNPs across 29 chromosomes. (**B**) Distribution of SNP counts across 29 chromosomes. (**C**) Functional classification of SNPs. (**D**) Minimum allele frequency distribution map. (**E**) Polymorphism information content distribution map.

This study utilized a 150K SNP chip to perform genome-wide SNP detection on 750 Xinjiang Brown cattle, resulting in a total of 94,173 SNP markers for subsequent analysis. Among the autosomes, chr 1 is the longest at 158.86 Mb and contains the highest number of SNP loci, totaling 5825. In contrast, chr 25 is the shortest at 42.77 Mb and has the fewest SNP loci, with only 1609 (Figure 1A,B). Functional annotation results (Figure 1C and Table S2) indicate these SNPs predominantly reside in intergenic regions (53,376, 56.68%) and intronic regions (37,086, 39.38%). Exons accounted for only 1.27% of total SNPs, comprising 617 non-synonymous SNPs and 511 synonymous SNPs. Genetic diversity in the Xinjiang Brown cattle population was assessed based on the high-quality SNPs identified. Results showed that the highest proportion of SNPs (29.75%) had a MAF between 0.3 and 0.4, while the lowest proportion (5.8%) had a MAF between 0 and 0.1. The average MAF was 0.276 (Figure 1D, Table 1). The polymorphic information content (PIC) of genomic SNPs ranged from 0.095 to 0.500, with an average PIC of 0.376. Among these, 13,277 SNPs (14.10%) had a PIC between 0 and 0.25. At the same time, 80,896 SNPs (85.90%) had a MAF between 0.25 and 0.5 (Figure 1E, Table 1). The observed average heterozygosity in the Xinjiang Brown cattle conservation population was 0.345, while the expected average heterozygosity was 0.376 (Table 1). The observed heterozygosity was slightly lower than the expected value, though the difference was not substantial. This discrepancy suggests potential selective pressure may be influencing the population.

### 3.2. Analysis of Genetic Distance Matrix and Kinship Matrix

**Figure 2:**
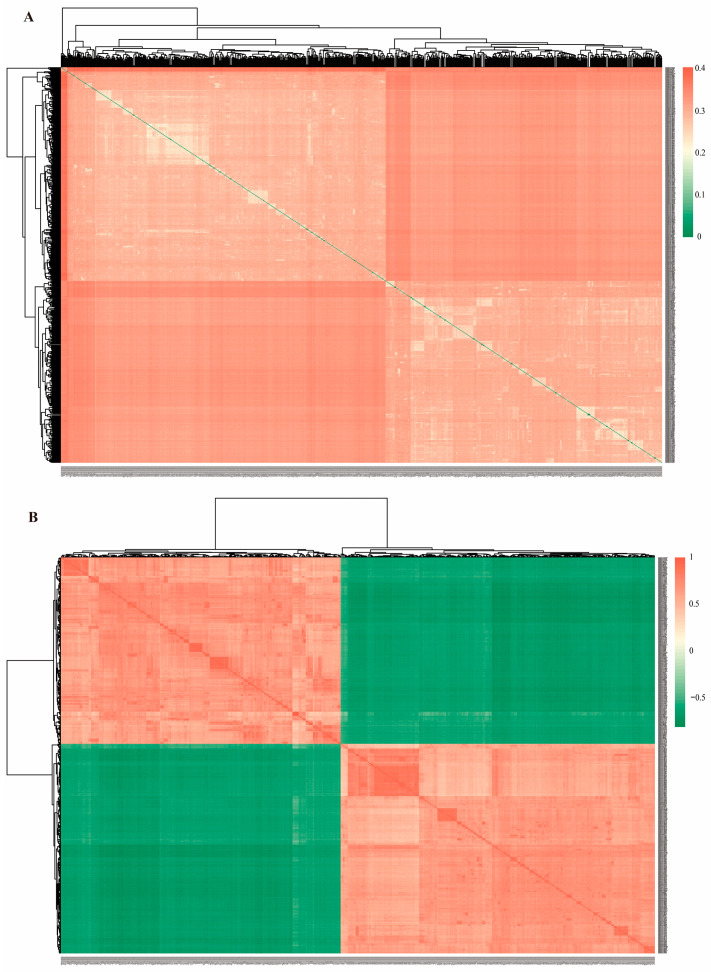
Visualization results of the distance matrix for Xinjiang Brown cattle. (**A**) IBS genetic distance matrix. (**B**) Phylogenetic G matrix. Note: The horizontal and vertical axes represent individual numbers of Xinjiang Brown cattle in this study. Each square in the IBS plot indicates the genetic distance between individuals; colors closer to red signify greater genetic distance, and vice versa. Each square in the G matrix represents the kinship relationship between individuals; colors closer to red indicate closer kinship, and vice versa.

Using Plink software to calculate IBS genetic distances among Xinjiang Brown cattle individuals, the results showed that the IBS distance values among 750 Xinjiang Brown cattle individuals ranged from 0.000021 to 0.406242, with an average IBS value of 0.313. This indicates that the average genetic distance between individuals is relatively large, with significant variation. The visualized IBS distance matrix for the Xinjiang Brown cattle population is shown in Figure 2A. Most pairs of Xinjiang Brown cattle individuals exhibited a relatively distant IBS genetic distance, indicating a moderate degree of kinship (represented by squares closer to red in Figure 2A). Among the 280,875 unique pairwise comparisons from the 750 Xinjiang Brown cattle, 192 pairs (0.068%) showed a closer IBS genetic distance, suggesting a closer kinship (IBS distance < 0.2), which corresponds to the squares closer to green in Figure 2A.

The results from the IBS genetic distance matrix show that most pairwise kinship estimates were moderate, as indicated by squares that are closer to green in Figure 2B. Out of 280,875 pairs analyzed, 186 pairs (0.066%) exhibited a closer kinship (G distance > 0.5), represented by squares closer to red in Figure 2B. Both results indicate that only a minimal number of closely related individual pairs exist within the Xinjiang Brown cattle population.

### 3.3. Principal Component Analysis and Linkage Disequilibrium Analysis

**Figure 3:**
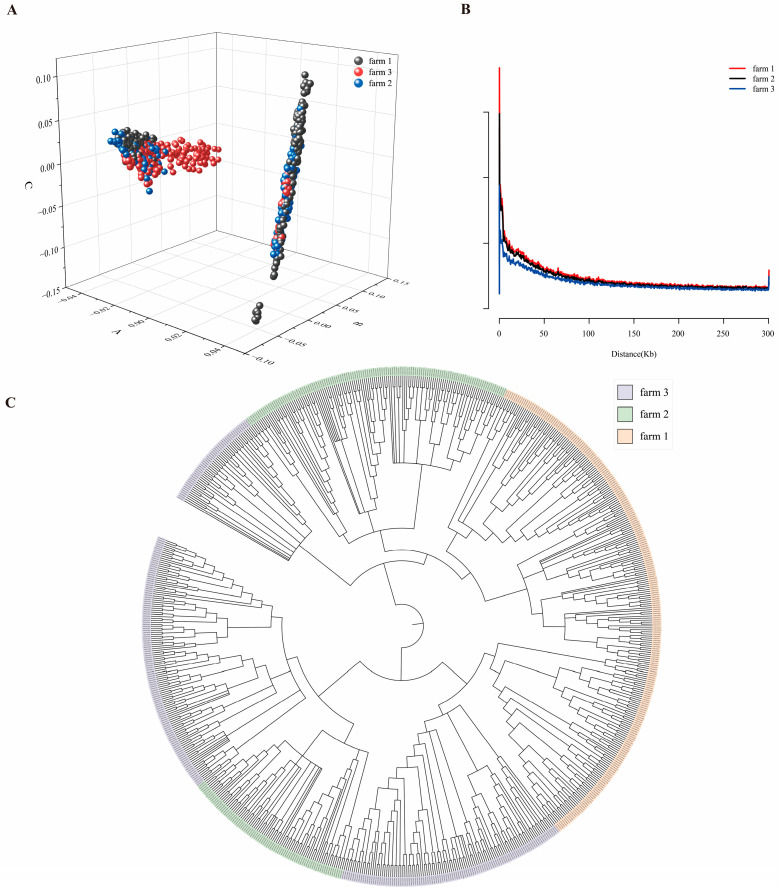
Visualization of the population structure of Xinjiang Brown cattle. (**A**) Principal Component Analysis. (**B**) Linkage Disequilibrium Decay Analysis. The horizontal axis represents the distance at which LD occurs; the vertical axis represents the LD correlation coefficient r^2^. (**C**) Phylogenetic Tree.

After quality control, 94,173 SNPs were retained for subsequent analysis. PCA results indicated overlapping populations among the three breeding farms, reflecting genetic connections between some individuals across these farms. Additionally, significant genetic distance existed within each breeding farm (Figure 3A). Figure 3B used PopLDdecay to calculate pairwise r^2^ values for the three breeding farm populations, comparing linkage disequilibrium (LD) levels among Xinjiang Brown cattle groups. The figure demonstrates differing rates of linkage disequilibrium decay among the three breeding farms. Xinjiang Brown cattle from farm 3 exhibited the fastest LD decay, followed by farm 2, while farm 1 showed the slowest decay rate. This indicates that farm 3 possesses the highest genetic diversity among Xinjiang Brown cattle; breeding farm 2 exhibits diversity second only to farm 3. Breeding farm 1 exhibited the slowest LD decay, with significantly lower genetic diversity compared to farms 2 and 3. The NJ tree constructed by MEGA software is shown in Figure 3C. The individuals from the three breeding farms did not form a completely independent branch. The clustering trend was generally consistent with the overall differentiation trend of the population in the PCA analysis, indicating a certain genetic relationship among the three breeding farm populations.

### 3.4. Inbreeding Coefficients Based on Pedigree

The pedigree inbreeding coefficients for Xinjiang Brown cattle across breeding farms are presented in Table 2. Among the average inbreeding coefficients, breeding farm 1 had the lowest at 0.0017, followed by farm 2 at 0.0189, with farm 3 having the highest at 0.0043. Both breeding farm 1 and farm 2 had maximum inbreeding coefficients of 0.25 and minimum coefficients of 0.125. Farm 3 exhibited a maximum inbreeding coefficient of 0.125 and a minimum of 0.0625. One-way ANOVA was used to test the difference in average *F_PED_* values among the three farms. The results showed that there was no statistically significant difference in *F_PED_* values between farms (*p* > 0.05).

### 3.5. Basic Statistics of Genome-Wide ROH

**Figure 4:**
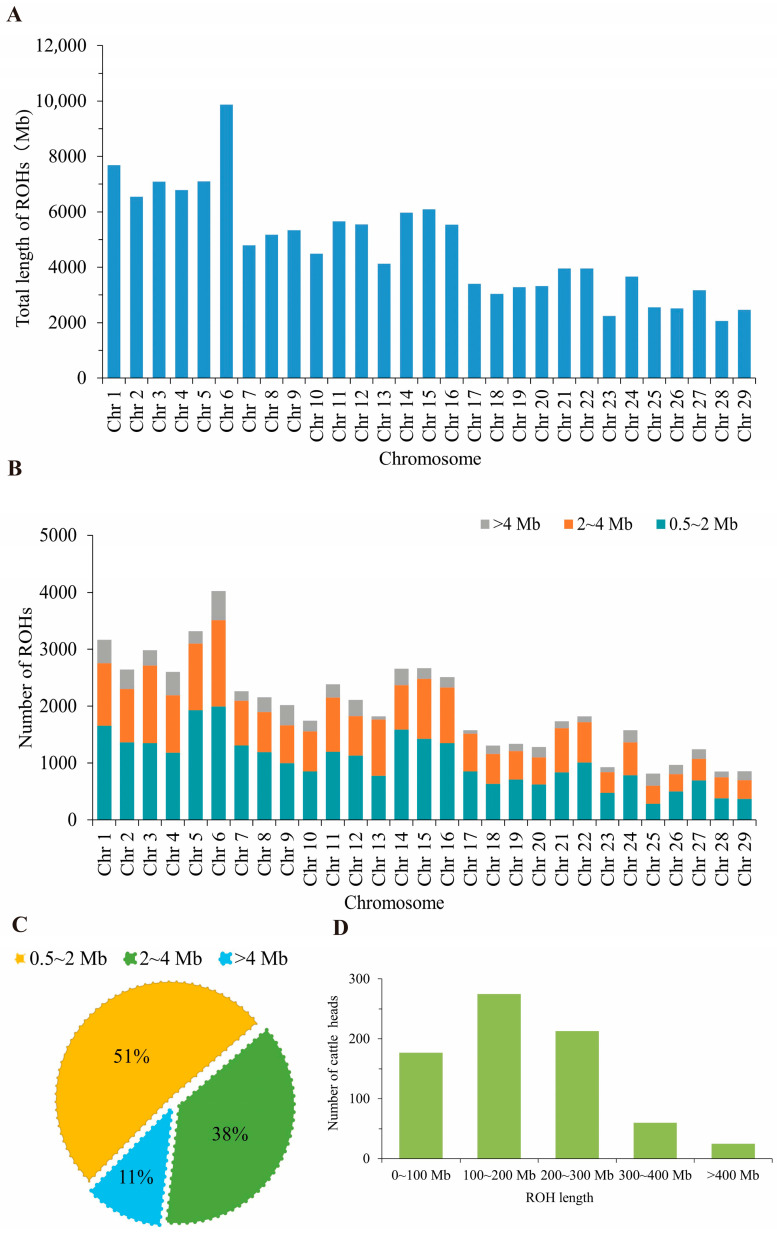
Basic statistics of ROH across the entire genome of Xinjiang Brown cattle. (**A**) Length distribution of ROH on autosomes. (**B**) Distribution of ROH per autosome across different physical length categories. (**C**) Proportion of ROH in different physical length categories. (**D**) Sample size distribution of individual ROH lengths.

A total of 57,381 ROH segments were detected in the Xinjiang Brown cattle population, with a cumulative ROH length of 134.20 GB. Each individual carried an average of 76.51 ROH segments, with an average ROH length per individual of 183.23 Mb and an average segment length of 2.39 Mb. As shown in Figure 4A,B, the longest ROH on chromosome 6 (9874.14 Mb) was observed in the Xinjiang Brown cattle population, followed by chromosome 1 (7681.59 Mb), while the shortest ROH was on chromosome 28 (2059.18 Mb). Among autosomes, chromosome 25 contained the fewest ROHs (814), while chromosome 6 harbored the most (4030), with 49.48% falling within the 0.5~2 Mb range. Figure 4C shows the proportion of ROHs across different physical length categories (0.5~2 Mb, 2~4 Mb, >4 Mb). ROHs in the 0.5~2 Mb range constituted the largest proportion at 51.31%, significantly exceeding other length categories. The second most common were those in the 2–4 Mb range (37.88%), while those with ROHs greater than 4 Mb represented the smallest proportion (10.81%). Figure 4D indicates that the majority of individuals had ROH lengths ranging from 150 to 200 Mb, while those with ROHs exceeding 400 Mb were the least common.

### 3.6. Genomic Inbreeding Coefficients

**Figure 5:**
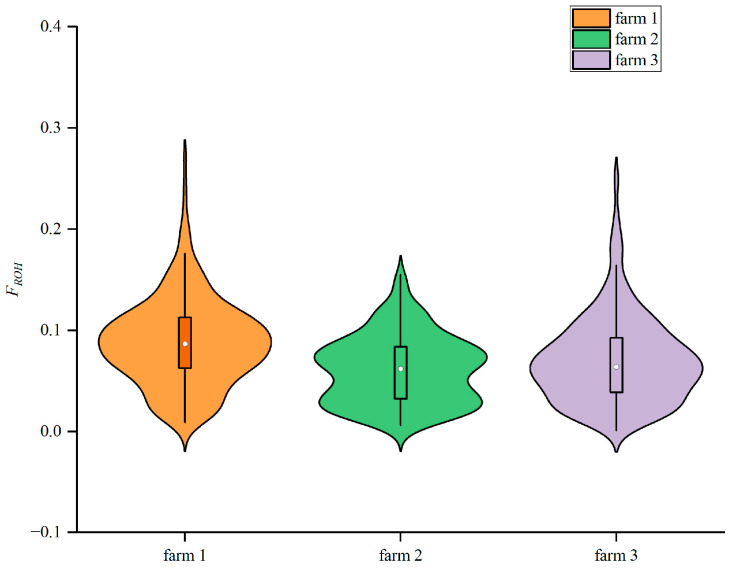
Inbreeding Coefficient of Xinjiang Brown cattle Based on ROH.

Table 3 and Figure 5 illustrate the distribution and visualization of genomic inbreeding coefficients across different breeding farms. Farm 1 exhibited coefficients ranging from 0.0090 to 0.2713, with an average of 0.0878. Breeding farm 2 inbreeding coefficients ranged from 0.0059 to 0.1552, with an average of 0.0609. Breeding farm 3 inbreeding coefficients ranged from 0.0013 to 0.2512, with an average of 0.0704.

### 3.7. Identification and Enrichment Analysis of Candidate Genes in High-Frequency ROH Regions

**Figure 6:**
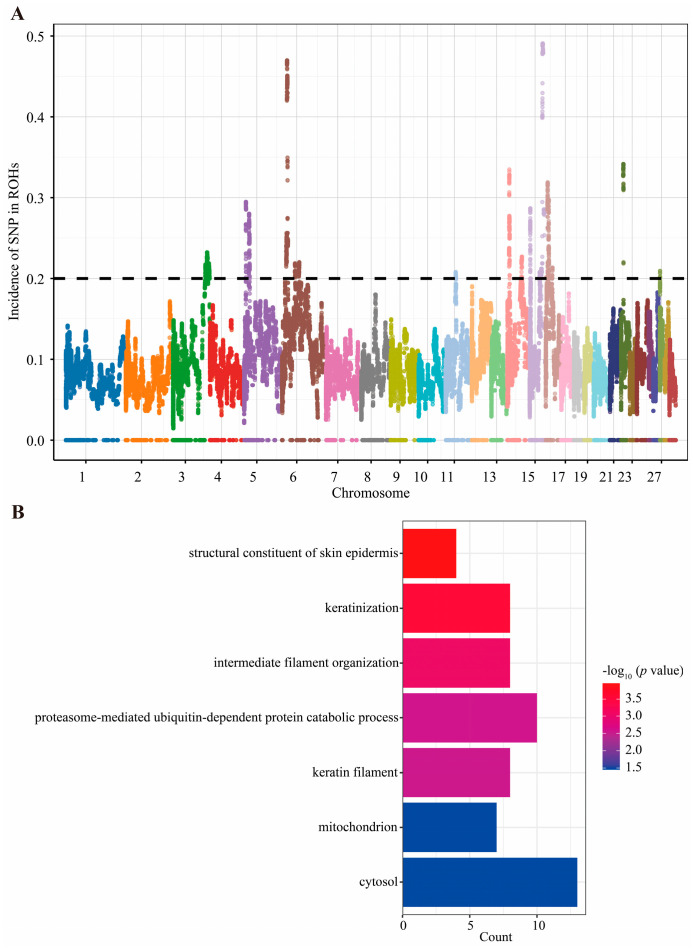
Identification and annotation of candidate genes in high-frequency ROH regions of Xinjiang Brown cattle. (**A**) Manhattan plot of SNP percentage within ROHs. (**B**) GO enrichment analysis of candidate genes.

The distribution of high-frequency ROH regions in the Xinjiang Brown cattle population is shown in Figure 6A. Chromosomes 3, 5, 6, 11, 14, 15, 16, 22, and 27 all exhibited peak counts exceeding the threshold, indicating potential enrichment of high-frequency ROH regions associated with inbreeding within these areas. Using a threshold of 0.2 for high-frequency ROHs, this study identified 1903 high-frequency ROH regions annotated to 61 genes (Table S3). Genes such as *LCORL*, *CHCHD7*, *FAM110B*, *NR4A1*, *PER2*, *SESN3*, and *PYCR2* may be associated with important economic traits of Xinjiang Brown cattle, including growth and body conformation, reproduction, meat quality, and milk production.

GO enrichment analysis revealed significant enrichment in seven pathways (Figure 6A and Table S4). Among biological process annotations (GO_BP), significantly enriched entries included: keratinization (*p* = 0.00029), intermediate filament organization (*p* = 0.000875), proteasome-mediated ubiquitin-dependent protein catabolic process (*p* = 0.00256). Significantly enriched entries in the cellular component annotation (GO_CC) included: keratin filament (*p* = 0.00282), mitochondrion (*p* = 0.035), and cytosol (*p* = 0.0352). Significantly enriched entries in molecular function annotation (GO_MF) included: structural constituent of skin epidermis (*p* = 0.000112). These enriched pathways are primarily involved in skin health, hair quality, cellular structural stability, stress resistance, energy metabolism, and other aspects. These pathways are associated with the environmental adaptability, growth and development, reproductive efficiency, and meat production performance of Xinjiang Brown cattle.

## 4. Discussion

The primary purpose of analyzing population genetic diversity is to investigate genetic variation within populations, thereby revealing key issues such as evolutionary history and environmental adaptability. It also provides crucial evidence for the sustainable development of population genetic resources [33,34]. Generally, higher population genetic diversity correlates with greater adaptability and survival capacity in response to environmental changes. MAF represents the estimated frequency of less common alleles at specific loci within a population [35]. In this study, the average MAF for the Xinjiang Brown cattle population was 0.276, slightly lower than the average MAF (0.28) calculated by Zhu et al. [36] in the Simmental cattle population. According to Botstein et al. [37], polymorphism levels can be categorized into three groups based on the PIC value: PIC > 0.5 indicates high polymorphism, 0.25 ≤ PIC ≤ 0.5 denotes moderate polymorphism, and PIC < 0.25 signifies low polymorphism. Most SNPs in this study fell within the 0.25~0.5 range, indicating moderate polymorphism sites. This suggests that the Xinjiang Brown cattle population possesses relatively rich genetic diversity and moderate genetic variation. Heterozygosity serves as a crucial indicator of genetic variation within a population, reflecting both genetic diversity and adaptive capacity. When Ho exceeds He, genetic diversity is abundant; conversely, a lower He suggests potential inbreeding within the population [38,39,40]. This study found that the observed heterozygosity of the Xinjiang Brown cattle population was lower than the expected heterozygosity, though the difference was not significant. This suggests that the population may have experienced some selective pressure, with genetic diversity influenced by factors such as geographic location and pasture management practices. The results indicate that the population maintains a certain level of genetic diversity and has not yet exhibited obvious inbreeding effects. LD analysis can be used to assess differences in population genetic diversity; a faster rate of LD decay indicates higher genomic genetic diversity within the population [41]. In this study, the Xinjiang Brown cattle population at breeding farm 3 exhibited faster LD decay than those at farms 1 and 2, indicating higher genetic diversity and lower genomic selection pressure in this population. The PCA results indicate some overlap among individuals, reflecting genetic connections between the three breeding farms. Overall, the genetic backgrounds of individuals from different farms show minimal differentiation.

Using breeding and calving records collected from three breeding sites in Xinjiang, the data underwent quality control before being analyzed for pedigree inbreeding coefficients. Based on pedigree-based inbreeding coefficients, the number of unique ancestors across all three farms was zero. The average *F*_PED_ values for breeding farms 1, 2, and 3 were 0.0017, 0.0189, and 0.0043, respectively, with farm 2 exhibiting a higher average *F*_PED_ than the other two farms. The average inbreeding coefficient across all farms was below the critical threshold for inbreeding depression, 0.0625 [42]. Makanjuola et al. reported average *F*_PED_ values of 0.0774 and 0.0720 for Holstein and Jersey cattle, respectively [43]. Chen et al. assessed average *F*_PED_ values of 0.0257, 0.0248, and 0.0268 for three Holstein dairy farms in Beijing [44], all of which were higher than the results of this study. Reza et al. calculated pedigree-based inbreeding coefficients for the entire Iranian Holstein population, yielding average *F*_PED_ values ranging from 0.0083 to 0.0168 [45], which are comparable to the results of this study. *F*_PED_ primarily utilizes pedigree information to assess individual inbreeding levels. However, its excessive reliance on the completeness and accuracy of pedigrees, coupled with the limited number of generations recorded, may fail to accurately reflect inbreeding among distant ancestors, potentially leading to underestimated inbreeding coefficients [46]. Leroy’s research revealed that inbreeding depression adversely affects livestock production performance, with each 0.01 increase in the inbreeding coefficient resulting in an average decline of 0.00137 in production performance [47]. Reducing inbreeding more effectively enhances the performance and health of offspring. Although the average inbreeding coefficients across all herds in this study were below the threshold for inbreeding depression, individual animals in some herds exhibited coefficients at or above this threshold. Future breeding programs should adjust mating strategies based on evaluation results to reduce inbreeding coefficients and optimize herd genetic structures.

ROH can reflect genetic diversity within a population, assess inbreeding history and levels, and more across the genome. In this study, the Xinjiang Brown cattle exhibited the highest number of ROH segments between 0.5 and 2 Mb, while the number exceeding 4 Mb was the lowest. This suggests that the Xinjiang Brown cattle population has not undergone significant inbreeding in recent times. Differences in ROH number and length distribution can reveal genetic diversity disparities between populations [48]. When breeders alter their selection criteria (e.g., prioritizing certain economic traits, such as milk yield or reproductive performance), they may exert greater selective pressure on specific genomic regions. Such shifts in selection pressure directly influence the abundance and length distribution patterns of ROHs [17]. Long ROHs indicate recent inbreeding, while short ROHs reflect more distant genetic relationships [49]. Results show short ROH fragments constitute the largest proportion of total length, suggesting inbreeding events in these three breeding farms occurred in the distant past. This reflects the breeding management achievements of recent years across these farms, which have effectively controlled the rise in inbreeding levels. Statistical analysis of ROH length, quantity, and distribution within the Xinjiang Brown cattle genome enables a more precise assessment of individual inbreeding levels, facilitating effective avoidance of inbreeding and promotion of genetic diversity in mating strategies. Keller et al. found that the inbreeding coefficient calculated based on ROH (*F*_ROH_) better reflects the true inbreeding level of a population [50] and more accurately indicates the degree of inbreeding depression [43]. The ROH-based inbreeding coefficient results show that the average inbreeding coefficients for breeding farms 1, 2, and 3 were 0.0878, 0.0609, and 0.0704, respectively. These values exceed the average *F*_ROH_ calculated from ROH for Simmental cattle (0.0003) and South African Nguni cattle (0.033) reported in previous studies [51,52], but are lower than those for Hereford cattle (0.229) [53], and similar to the average *F*_ROH_ values for Kazakh White-headed cattle (0.084) and Holstein cattle (0.079) [54,55]. This discrepancy may stem from differences in breed characteristics and population size among the groups, as well as variations in chip density during individual cattle genotyping, which can also influence ROH detection results [56]. Breeding farm 2 exhibited a lower inbreeding coefficient compared to farms 1 and 3, indicating a reduced risk of inbreeding depression. Previous studies have demonstrated that inbreeding assessed based on ROH reflects both recent and distant generations of inbreeding, providing a more comprehensive representation of an animal’s inbreeding level [57]. In future breeding programs, farms with high inbreeding coefficients should avoid further inbreeding and minimize practices that exacerbate it to prevent trait deterioration. Conversely, farms with low inbreeding coefficients should monitor for excessive outcrossing and strengthen purebred selection to prevent the loss of genetic resources. Breeding farms can optimize breeding strategies by monitoring *F*_ROH_ [58], thereby reducing inbreeding and maintaining genetic diversity while enhancing herd productivity and adaptability.

High-frequency ROH regions in the genome correspond to genomic segments that may have been subject to selective pressure or inbreeding effects during the population’s history. In this study, 61 candidate genes were identified in the high-frequency ROH region of the Xinjiang Brown cattle genome. These genes are closely related to important economic traits such as reproduction, growth, lactation, and environmental adaptation, reflecting the genomic characteristics left by the population during historical selection or inbreeding. Among them, multiple genes play key roles in reproduction, growth, and development. For example, *LCORL*, a transcription factor, is not only involved in testicular spermatogenesis [59] but is also significantly associated with body height and cross height [60]. *CHCHD7* is associated with body shape traits in Angus cattle and German Fleckvieh cattle [61,62], while *CWC15* affects the reproductive traits of Jersey cattle [63]. *FAM110B* is particularly worthy of attention. This gene is not only associated with sexual precocity and the first calving age in Brahman cattle [64], but also plays an important role in growth and development, carcass weight, and meat quality in beef cattle [65]. In terms of lactation and milk composition synthesis, *DTX4* is involved in the regulation of milk fat synthesis in bovine mammary epithelial cells [66], and *NR4A1* is significantly associated with milk yield and milk composition traits in Chinese Holstein cattle [67]. *PER2* affects fatty acid synthesis and desaturation by participating in milk fat metabolism [68], and *KRT7* provides a new molecular perspective on the function of mammary epithelial cells [69]. In addition, some genes are related to energy metabolism, immunity, and stress adaptation, reflecting the physiological regulatory mechanisms of Xinjiang Brown cattle in specific environments. In addition to affecting lactation, *NR4A1* is involved in immune regulation and energy metabolism and plays a role in macrophage anti-inflammatory responses and glucose and lipid metabolism [70,71]. As a stress-sensitive gene, *SESN3* can regulate lipid metabolism and is related to fat deposition in grazing yaks [72]. *LEMD3* is involved in adipose tissue development [73], *PYCR2* responds to inflammation and oxidative stress, and is associated with immune-inflammatory traits [74]. *TMEM33* is also found to be associated with heat-stress physiological indicators in Holstein cattle [75].

## 5. Conclusions

This study conducted a population genetic analysis of Xinjiang Brown cattle from three breeding farms in the Xinjiang region. The results revealed that all three populations exhibit relatively high genetic diversity, with breeding farm 3 showing the highest level of diversity. The average pedigree-based inbreeding coefficient (*F*_PED_) was low across all farms. Additionally, the average genomic inbreeding coefficient calculated from runs of homozygosity (*F*_ROH_) was consistently higher than the average *F*_PED_. This suggests that *F*_ROH_ provides a more accurate assessment of inbreeding levels by identifying ROH segments in the Xinjiang brown cattle genome, offering advantages over *F*_PED_. In regions with high-frequency ROH, we identified 61 candidate genes, including *LCORL*, *FAM110B*, *NR4A1*, and *PER2*, which may be associated with important economic traits such as growth, body conformation, meat quality, and milk production in Xinjiang Brown cattle. These findings are expected to lay the groundwork for genetic resource conservation and molecular marker-assisted breeding.

## Figures and Tables

**Table 1:** Statistical Parameters Related to Genetic Diversity in Xinjiang Brown cattle.

Breed	Number of SNPs	MAF	PIC	He	Ho
XJBC	94,173	0.276	0.376	0.376	0.345

**Table 2:** Inbreeding coefficient of spectrum.

Classify	Farm 1	Farm 2	Farm 3
Average inbreeding coefficients	0.0017	0.0189	0.0043
Maximum of inbreeding coefficients	0.25	0.25	0.125
Minimum of inbreeding coefficients	0.125	0.0625	0.0625

**Table 3:** Genomic inbreeding coefficient.

Classify	Farm 1	Farm 2	Farm 3
Average inbreeding coefficients	0.0878	0.0609	0.0704
Maximum of inbreeding coefficients	0.2713	0.1552	0.2512
Minimum of inbreeding coefficients	0.0090	0.0059	0.0013

## Data Availability

The data and material used in this research are available from the corresponding author on request.

## Supplementary Materials

The following supporting information can be downloaded at: https://www.mdpi.com/article/10.3390/ani16010042/s1, Table S1: Distribution of 750 Xinjiang Brown cattle; Table S2: Functional classification of the detected SNPs; Table S3: Candidate Genes for Regional High-Frequency ROH in Xinjiang Brown cattle; Table S4: GO Enrichment Analysis of Xinjiang Brown cattle.
