# Supplementary material for: Analysis of Inbreeding Coefficient and Genetic Diversity in Xinjiang Brown Cattle Based on Pedigree and ROH Evaluation
Source: Animals (Basel). 2025 Dec 23;16(1):42. doi: 10.3390/ani16010042 (PMC12784852; doi:10.3390/ani16010042)
Supplement: Supplementary file 1 [file animals-16-00042-s001.zip › animals-4006139-supplementary.pdf]

Analysis of Inbreeding Coefficient and Genetic Diversity in Xinjiang Brown Cattle Based on Pedigree and ROH Evaluation

Supplementary Table S1 Distribution of 750 Xinjiang Brown cattle

Serial No.	Farm ID	Breeding farm Name	Sample Size (n)
1	farm 1	Xinjiang Yanben Brown cattle Breeding and Development Co., Ltd.	254
2	farm 2	Tacheng Agriculture and Animal Husbandry Technology Co., Ltd.	211
3	farm 3	Yili New Brown cattle Breeding farm	285

Supplementary Table S2 Functional classification of the detected SNPs

Functional classification	Number of SNPs
intergenic	53376
intronic	37086
upstream	616
downstream	629
UTR3	905
UTR5	269
splicing	38
ncRNA	11
exonic	1196
other	47
nonsynonymous	617
synonymous	622
stopgain	46
stoploss	2
unknown	20

Supplementary Table S3 Candidate Genes for Regional High-Frequency ROH in Xinjiang Brown cattle

Serial Number	Gene	Serial Number	Gene	Serial Number	Gene
1	CHRM4	24	LOC524768	47	CLOCK
2	AMBRA1	25	NR4A1	48	TMEM33
3	MADD	26	SESN3	49	MIXL1
4	AGBL2	27	LOC101905683	50	REST
5	NUP160	28	LOC787600	51	IGFBP7
6	EOGT	29	KRT7	52	KIF26B
7	LOC107131675	30	MSRB3	53	THAP4
8	LBR	31	PLD5	54	LOC112448807
9	DNAH14	32	LCORL	55	KRT82
10	SLC4A8	33	UBE2F	56	FBXO48
11	WDR26	34	CHCHD7	57	TNN
12	SCN8A	35	PER2	58	COL6A3
13	CSRNP2	36	FAM110B	59	STUM
14	LOC104974344	37	KRT81	60	RABGAP1L
15	LOC784993	38	LEMD3	61	CACYBP
16	LOC104974345	39	LOC112446047		
17	KMO	40	LOC112446048		
18	DTX4	41	LOC107132360		
19	SELENOH	42	ANKMY1		
20	CTNND1	43	AQP12B		
21	AMOTL1	44	PYCR2		
22	CHML	45	COP1		
23	CWC15	46	ANO7		

Supplementary Table S4 GO Enrichment Analysis of Xinjiang Brown cattle

Category	Term	Count	PValue
MF	structural constituent of skin epidermis	4	0.000112
BP	keratinization	4	0.00029
BP	intermediate filament organization	4	0.000875
BP	proteasome-mediated ubiquitin-dependent protein catabolic process	5	0.00256
CC	keratin filament	4	0.00282
CC	mitochondrion	7	0.035
CC	cytosol	13	0.0352
